# Association of *Fusobacterium nucleatum* infection with colorectal cancer in Kazakhstani patients

**DOI:** 10.3389/fonc.2024.1473575

**Published:** 2024-12-12

**Authors:** Gulmira Kulmambetova, Botakoz Kurentay, Alua Gusmaulemova, Talgat Utupov, Dana Auganova, Pavel Tarlykov, Meiram Mamlin, Saule Khamzina, Sanzhar Shalekenov, Arman Kozhakhmetov

**Affiliations:** ^1^ Department of Genomics, National Center for Biotechnology, Astana, Kazakhstan; ^2^ Multidisciplinary Surgery Center, National Research Oncology Center, Astana, Kazakhstan; ^3^ Department of Surgery, Nazarbayev University School of Medicine, Astana, Kazakhstan

**Keywords:** abundance, colorectal cancer, Fusobacterium nucleatum, Kazakhstan, qPCR

## Abstract

**Objectives:**

*Fusobacterium nucleatum* is a gram-negative anaerobic bacillus associated with colorectal cancer (CRC). We aimed to determine the abundance of *F*. *nucleatum* and other CRC-associated bacteria using quantitative real-time polymerase chain reaction (qPCR) analysis to detect the possible correlations between tumor and normal tissues and the relationships between patients’ clinical characteristics, diet, and CRC-associated bacteria.

**Methods:**

A total of 249 biopsy samples of tumor and paired normal tissues were collected from patients with CRC. Biopsy samples were screened for detection of *F. nucleatum* using qPCR targeting *nusG* gene. *Bacteroides fragilis*, *Escherichia coli*, and *Streptococcus gallolyticus* were also detected in the samples using species-specific genes.

**Results:**

The frequencies of detection of *F. nucleatum* in the tumor and normal tissues of patients with CRC were 43.37 and 24.1%, respectively (P < 0.05). Statistical analysis using cycle threshold (Ct) values from qPCR data and clinical characteristics showed that tumor size, tumor location, and processed meat consumption were significantly correlated with the abundance of *F. nucleatum* (P < 0.05). The significance of the prevalence of *B. fragilis* and *E. coli* in tumor tissues was marginally higher than that in normal tissues (P < 0.1), and the consumption of processed/red meat affected the prevalence of these bacteria (P < 0.05).

**Conclusions:**

Our results showed an association between the presence of *F. nucleatum* in tumor tissues and CRC, indicating that *F. nucleatum* may be a potential marker for CRC diagnosis. *F. nucleatum* is enriched in CRC tissues and is associated with CRC development.

## 1 Introduction

*Fusobacterium nucleatum* is a gram-negative anaerobic bacillus present in the oral microbiota and is associated with colorectal cancer (1, 2). CRC is the third most common cancer and the second leading cause of cancer-related mortalities worldwide. *F. nucleatum* has gained attention in recent years because of its potential role in CRC development (3, 4). Various risk factors influence the development of cancer, including age, family history of the disease, inherited genetic conditions (such as Lynch syndrome and familial adenomatous polyposis), personal history of inflammatory bowel disease (such as Crohn’s disease or ulcerative colitis), obesity, physical inactivity, smoking, heavy alcohol consumption, and a diet high in red and processed meats and low in fiber. Studies have shown that dietary patterns play a significant role in the development of colorectal cancer (5). Certain diets, identified through the empirical dietary inflammatory pattern (EDIP) assessment, have been linked to increased intestinal inflammation and a higher risk of *F. nucleatum*-positive colorectal carcinomas (6). Diet-induced intestinal inflammation alters the gut microbiome, promoting colorectal carcinogenesis. A high consumption of red and processed meats has been associated with an increased risk of colorectal cancer, potentially due to carcinogens such as nitrates, nitrites, and heterocyclic amines (7). Environmental factors, including dietary habits and antibiotic use, may also affect the behavior of *F. nucleatum* in the colon. On the other side, the roles of intestinal microorganisms in initiating and promoting the development of colorectal cancer are becoming increasingly well understood. There is a complex relationship between gut microbiota and colorectal cancer. Recent research has identified *Streptococcus gallolyticus*, enterotoxigenic *B. fragilis*, *F. nucleatum*, and *E. coli*, as potential pathogens associated with colorectal cancer (8). Although intestinal microbiota varies among individuals, certain bacterial species have been consistently linked to colorectal cancer. *S. gallolyticus*, a gram-positive cocci, is a reported risk factor for CRC (9). Enterotoxigenic *B. fragilis* (ETBF), which produces *B. fragilis* toxin (BFT), is known to cause diarrhea and contribute to inflammatory bowel disease (IBD) (10). Similarly, *E. coli*, a gut commensal bacterium, has been found to colonize the colonic by mucosa-associated *E. coli* at higher levels in colorectal cancer patients compared to healthy individuals (11, 12). However, the response to these risk factors may vary depending on the ethnicity and geographical location, thereby affecting the distribution and prognosis of CRC.

Although *F. nucleatum* is a common inhabitant of the human oral cavity, its abundance is elevated in colorectal tumors and adjacent tissues of patients with CRC (13, 14). Several studies have suggested a potential link between *F. nucleatum* and CRC (1, 15). This bacterium has been reported to promote inflammation, impair immune responses, alter tumor microenvironment, promote resistance to chemotherapy, and facilitate tumor growth and metastasis in preclinical models (16, 17). Additionally, *F. nucleatum* has been associated with a poor prognosis in patients with CRC (18). The presence of *F. nucleatum* in colorectal tissues has led to an interest in its potential as a diagnostic marker or therapeutic target for CRC. However, the role of *F. nucleatum* in the development of CRC remains unclear, for several reasons.

The gut microbiome is highly complex, comprising a diverse range of microorganisms. While *F. nucleatum* is more abundant in the tumors of some colorectal cancer (CRC) patients, its presence alone may not be sufficient to initiate cancer. The interactions between *F. nucleatum*, other microbes, and the host immune system may affect its potential role in cancer development, making it difficult to determine its precise contribution. While studies have linked *F. nucleatum* to colorectal cancer, it remains unclear whether the bacterium directly causes cancer development or if its presence results in changes in the tumor microenvironment. Whether *F. nucleatum* is a driver or merely a bystander in colorectal cancer continues to be an area of active investigation. Further research is needed to fully understand its role in CRC development, explore the clinical implications, and clarify the role of *F. nucleatum* — as a target, a biomarker, or a secondary by-product of tumor development. However, to our knowledge, the effect of local diet, demographics, and clinical characteristics of patients on the specific gut bacteria, comprising *F. nucleatum*, has not been investigated thoroughly in Kazakhstan.

In this study, we aimed to determine the abundance of *F. nucleatum* and other CRC-associated bacteria using quantitative real-time polymerase chain reaction (qPCR) analysis to detect the possible correlations between tumor and normal tissues, as well as relationships between patients’ clinical characteristics, diet, with CRC-associated bacteria.

## 2 Materials and methods

### 2.1 Patients

A total of 83 patients with histologically confirmed colorectal adenocarcinoma (39 men and 44 women; age range, 26–86 years) who underwent surgical resection at the National Research Oncology Center, Astana, Kazakhstan, between October 2022 and April 2024 were included in this study. Patients who had other oncological diseases, received preoperative radiation or chemotherapy, and/or had distant metastases were excluded. Biopsies were obtained from carcinoma tissues (CTs), adjacent normal tissues (ATs), and distant normal tissues (NTs, 10 cm beyond the cancer margins) of patients with CRC. In total, 249 tissue biopsy samples were collected in tubes containing 20% sucrose. Culture and DNA extraction for qPCR were performed within 2 h of tissue collection, and the remaining tissues were stored in a deep freezer (-80 °C) until use. All 83 patients were included in the qPCR study.

### 2.2 Ethics approval

The study protocol complied with the Declaration of Helsinki and was approved by the local ethics committee of the National Center for Biotechnology of the Ministry of Health of the Republic of Kazakhstan (Extract from Protocol No. 1, dated 04/01/2022). All methods were performed according to the relevant guidelines and regulations. Written informed consent was obtained from all participants.

### 2.3 Detection of CRC-associated bacteria using qPCR

DNA was extracted from the colon tissue samples using the QIAamp DNA Micro Kit (Qiagen, Germany) according to the manufacturer’s instructions. The DNA concentration and purity were recorded using a NanoDrop spectrophotometer (NanoDrop 1000; Thermo Fisher Scientific, USA). Specific genes were amplified by qPCR using a CFX384 Touch Real-Time PCR Detection System (Bio-Rad, USA) to detect four CRC-associated bacteria, namely, *F*. *nucleatum*, *Bacteroides fragilis*, *Escherichia coli*, and *Streptococcus gallolyticus.* The reaction mixture consisted of 5 μL SYBR Green (Biolabmix, Russia), 0.5 μL each of the specific primer pair (10 μM), and 50 ng/μL DNA template in a total reaction volume of 10 μL. *F. nucleatum* subsp. *nucleatum* (accession no. SRR24390575) and three clinical isolates (enterotoxigenic *B. fragilis* [ETBF], *E. coli*, and *S. gallolyticus*) were used for qPCR quality control. The clinical isolates were identified using matrix-assisted laser desorption/ionization time-of-flight (MALDI-TOF) mass spectrometry and *16S rRNA* sequencing. The cycle threshold (Ct) values for *F. nucleatum* and other bacteria were normalized to the amount of human DNA in each reaction mix using a primer set for the reference gene, the solute carrier organic anion (*SLCO*) transporter, as described previously (19). All assays were performed in duplicates, and the results were averaged. The fold changes of *F. nucleatum* abundance in diseased tissues over that in the matched normal colorectal tissues was calculated as 2^-ΔΔCt^.

Previously published primers with the following sequences were used: *F. nucleatum* forward primer, 5′-ACCCTCGTGTATGGTATGAAGT-3′; *F. nucleatum* reverse primer, 5′-TCAGCAACTTGTCCTTCTTGA-3′ (19); *SLCO* forward primer, 5′-ATCCCCAAAGCACCTGGTTT-3′; *SLCO* reverse primer, 5′-AGAGGCCAAGATAGTCCTGGTAA-3′ (19). The following primers were used to detect specific bacteria: *Bacteroides* forward primer, 5′-GGACATTTGGGAGTTCAGGAC-3′; *Bacteroides* reverse primer, 5′-TGCTTTTCTGATCTCTTCGGC-3′; *Streptococcus* forward primer, 5′-GGGAATTGTTATCGCCTGAA-3′; *Streptococcus* reverse primer, 5′-GTGCCAAAATTGGTGCTTTT-3′; *E. coli* forward primer, 5′-CTGATAGCGCGTGACAAAAA-3′; *E. coli* reverse primer, 5′-GGCACAGCACATCAAAGAGA-3′.

### 2.4 Statistical analysis

*F. nucleatum* levels determined by qPCR are given as 2^−ΔCt^, where ΔCt is the median of the difference in Ct between the test and reference genes. This relative quantification (RQ) was log-transformed to be analyzed as log2(1/2^−ΔCt^). The ratio of *F. nucleatum* levels between tumor and matched normal colorectal tissues is given as the fold increase, 2^−ΔΔCt^, where ΔΔCt is the median of the difference between ΔCt of diseased and ΔCt of normal tissues.

All statistical analyses were performed using the R software (https://www.r-project.org, version 4.2.0; the RStudio 2022.02.2). Continuous data are expressed as medians (25^th^ percentile, 75^th^ percentile) that were calculated using Student’s t-test of independent or paired samples. The Mann–Whitney (Wilcoxon) test was used to compare the results for two non-paired groups. The Kruskal–Wallis test was used to compare the median levels of *F. nucleatum* between more than two groups, such as different cancer stage subgroups. Categorical variables were analyzed using Fisher’s exact test. A value of P < 0.05 was defined as statistically significant, and a P-value between 0.05 and 0.1 was considered marginally significant.

## 3 Results

### 3.1 Clinical characteristics of patients

The clinical features of 83 patients with CRC and 249 biopsy samples were examined (**Supplementary Table S1**).

### 3.2 Frequency of occurrence of CRC−associated bacteria as determined using qPCR

The prevalence of the four CRC-associated bacteria was examined in the CTs, ATs, and NTs of patients with CRC using qPCR (**Table 1**). *F. nucleatum* was most frequently detected in CTs and slightly less frequently in ATs compared to that in the NTs of patients with CRC (43.4, 27.7, and 24.1%, respectively; P *=* 0.02). However, marginally significant differences in the prevalences of *E. coli*, and *B. fragilis* in CTs were higher than in ATs, and NTs of patients with CRC (P = 0.07, and P = 0.06, respectively). No significant difference in the prevalence of *S. gallolyticus* was observed among the tissues of patients with CRC.

**Table 1:** Frequency of colorectal cancer (CRC)-associated bacteria, as determined by quantitative real-time polymerase chain reaction (qPCR).

Bacteria	No. (%) of patients positive for qPCR			P value^a^
	CT (n=83)	AT (n=83)	NT (n=83)	
*Fusobacterium nucleatum*	36 (43.37)	23 (27.71)	20 (24.10)	0.02*
*Bacteroides fragilis*	40 (48.19)	31 (37.35)	25 (30.12)	0.06
*Escherichia coli*	57 (68.67)	53 (63.86)	43 (51.81)	0.07
*Streptococcus gallolyticus*	3 (3,61)	0 (0)	1 (1.20)	0.33

a P-values were calculated using Fisher’s exact test for count data. ^*^Statistically significant at P < 0.05.

### 3.3 Correlation between *F. nucleatum* infection and clinical characteristics of patients with CRC

**Figure 1:**
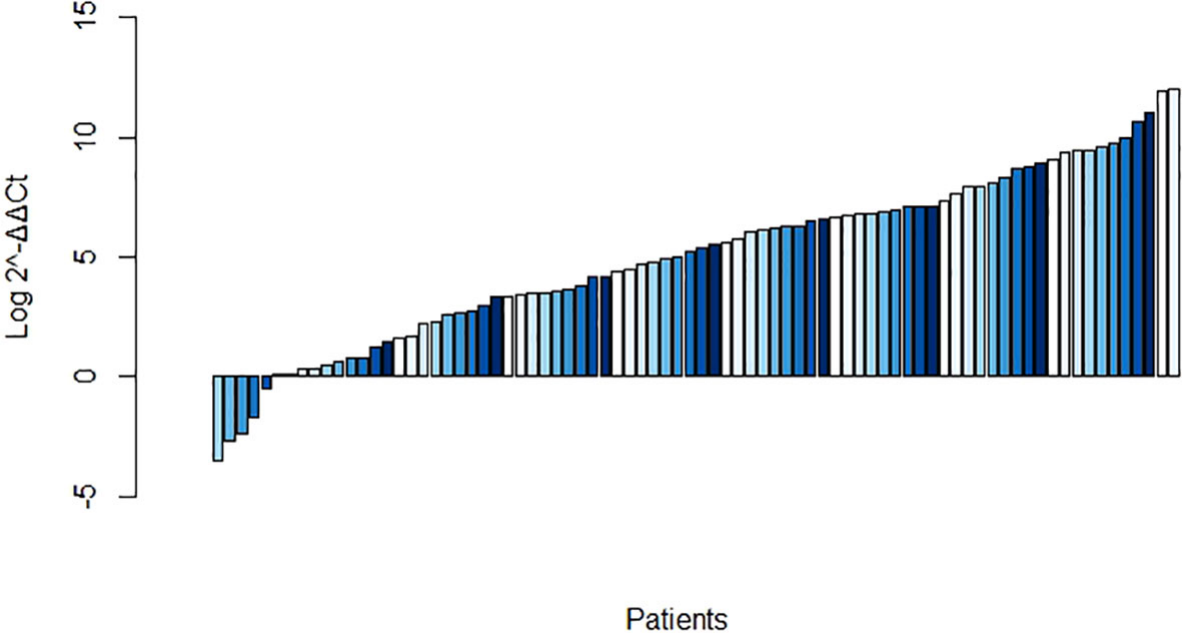
Log abundance of *Fusobacterium nucleatum* in colorectal cancer (CRC) tissues compared to that in matched normal tissues (n = 83).

Compared with that in the matched normal tissues, the *F. nucleatum* load was significantly overrepresented in 75 of 83 (90.36%) CRC samples (**Figure 1**). The median abundance of *F. nucleatum*, as determined by 2^-ΔΔCt^, was significantly greater in the tumor samples (19.4 [2.4–326.7]) than that in the matched normal controls (4.39 [0.99–28.26]; P = 0.001).

The associations between the clinical variables of the patients and *F. nucleatum* infection are summarized in **Table 2**. In total, 58 of the 83 (69.9%) CRC cases were localized in the distal part of the large intestine. The *F. nucleatum* level, expressed as fold changes (2^-ΔΔCt^; cancer versus normal tissues), in the distally located CRC (390.76 [28.78–3062.55]) was significantly higher than that in the proximally located CRC (27.19 [4.42–427.08]; P < 0.05). Distally located CRC was observed in 53 out of 75 (70.7%) patients with *F. nucleatum* over-abundance (fold change > 1) and five out of eight (62.5%) patients with *F. nucleatum* under-abundance (fold change < 1; P > 0.05). No significant association was observed between *F. nucleatum* infection and other clinical variables, such as patients’ sex, age, pathological differentiation, infiltration depth, lymph node metastasis, and cancer stage (P > 0.05; **Table 2**).

**Table 2:** Association of *Fusobacterium nucleatum* infection with clinicopathological variables in a cohort of 83 patients.

	n	Fold changes between cancer and normal tissues [2-ΔΔCt (median)]	P value
Gender			
Male Female	39 44	149.55 (4.64 - 1360.26) 175.76 (27.72 - 1056.83)	0.08
Age (yr)			
<65 ≥65	45 38	178.59 (5.21 - 2834.86) 98.03 (10.53 - 959.24)	0.75
Location of CRC			
Proximal Distal	25 58	27.19 (4.42 - 427.08) 390.76 (28.78 - 3062.55)	0.03^*^
Differentiation			
Moderately and high 2 Low 3	32 51	37.28 (4.76 - 1027.88) 250.97 (14.89 - 1201.12)	0.72
Tissue infiltration			
T1 + T2 T3 + T4	15 67	23.20 (3.16 - 1390.43) 231.4 (10.7 - 1145.8)	0.49
Lymph node metastasis			
N0 N1 + N2	46 37	184.17 (10.19 - 2610.87) 149.55 (9.65 - 976.88)	0.22
Stage			
I II III IV	11 34 36 1	27.19 (9.19 - 1599.99) 412.34 (10.36 - 2828.17) 164.07 (8.12 - 986.53) 84.76 (84.76 - 84.76)	0.99

^*^Statistically significant at P < 0.05.

### 3.4 Prevalence of *F. nucleatum* across different tumor stages and tissue types

**Figure 2:**
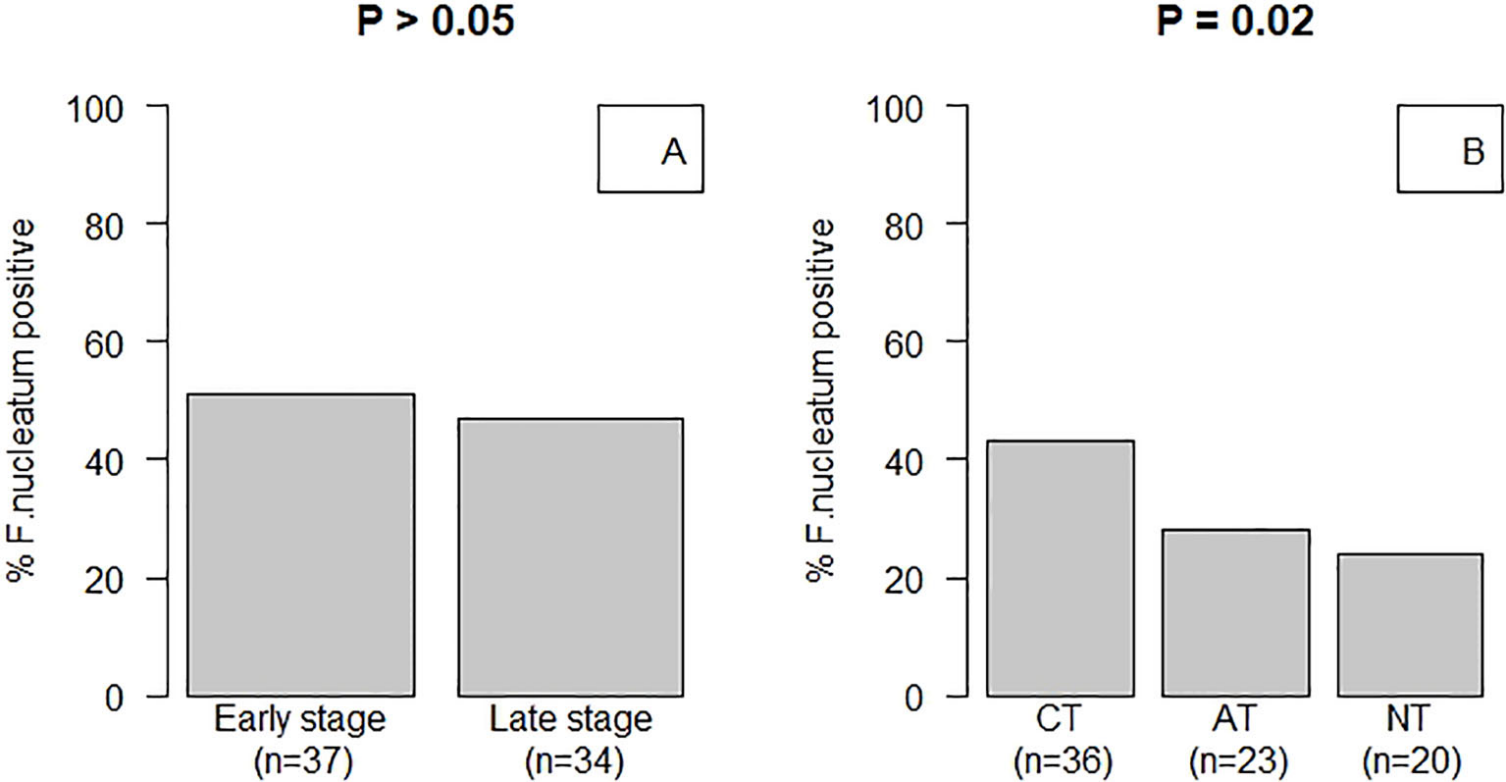
*Fusobacterium nucleatum* infection status of patients with CRC. **(A)** Patients with CRC were classified based on tumor stage: stages I/II were classified as early stage, and stages III/IV were classified as late stage. The prevalence of *F*. *nucleatum* was not significantly different between patients with early and late stage CRC (51 vs 47%, respectively; Fisher’s exact test, P > 0.05). **(B)** Carcinoma tissue (CT), adjacent normal tissue (AT), and normal tissue (NT) samples were collected from non-CRC sites from patients with CRC. *F*. *nucleatum* was significantly more prevalent in CTs (43.4%) compared to that in ATs (27.7%) and NTs (24.1%) of patients with CRC (pairwise Fisher’s exact test: CT vs. AT, P = 0.08; CT vs. NT, P = 0.04; AT vs. NT, P = 0.72). Additionally, *F*. *nucleatum* was observed in significantly higher numbers in CTs compared to that in NTs of patients (P < 0.02).

We examined the relationship between *F. nucleatum* positivity and clinicopathological features of patients with CRC. Patients with CRC were categorized according to tumor stage as early stage (I/II) or late stage (III/IV). *F. nucleatum* was detected at similar frequencies in both the early (51%) and late (47%) stages; however, this difference was not statistically significant (**Figure 2A**). Regarding tissue type, the prevalence of *F. nucleatum* was significantly higher in CTs (43.4%) compared to that in ATs (27.7%) and NTs (24.1%) in patients with CRC. No significant differences were observed between the AT and NT groups (**Figure 2B**). In *F. nucleatum*-positive cases, the Ct values obtained by qPCR were significantly lower in CTs than those in other tissue types (Mann–Whitney U test; P < 0.0001).

### 3.5 qPCR analysis

**Figure 3:**
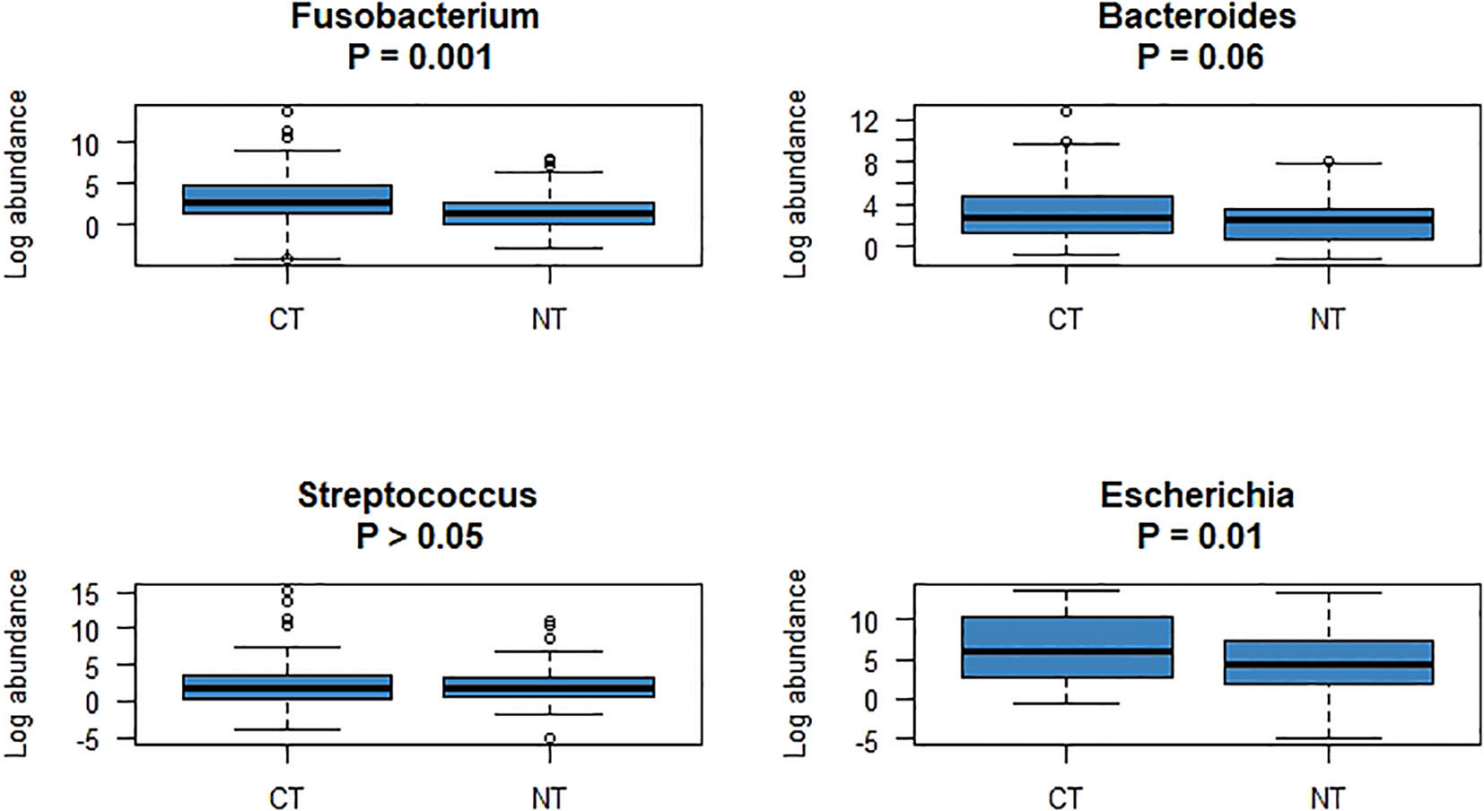
The abundances of four bacteria, which were significantly different between CTs and NTs, were compared using R software.

Duplicate qPCR assays were conducted for detection of the four bacterial genera in the three types of CRC tissue samples. In total, 996 Ct values were generated and used for further statistical analyses. Comparison of CTs with normal tissues of patients with CRC revealed that the Ct values for *Fusobacterium* and *Escherichia* were significantly different after false discovery rate correction for multiple testing (**Figure 3**). Regarding the clinical characteristics with continuous values, tumor sizes in patients with *Fusobacterium*-positive CRC were significantly larger than those in patients with *Fusobacterium*-negative CRC (4.75 ± 2.33 vs. 3.27 ± 1.92, respectively; P = 0.04); similarly, tumor sizes in patients with *Bacteroides*-positive CRC were significantly larger than those in patients with *Bacteroides*-negative CRC (4.66 ± 2.28 vs. 1 ± 0, respectively; P *=* 2.2e - 16; **Table 3**). Importantly, patients with *Fusobacterium*-positive CRC consumed significantly higher amounts of processed meat than patients with *Fusobacterium*-negative CRC (31.64 ± 47.76 vs. 8.75 ± 18.08, respectively; P = 0.02; **Table 3**). Moreover, patients with *Bacteroides*-positive CRC consumed significantly higher amounts of processed, red, and total meat than patients with *Bacteroides*-negative CRC (29.32 ± 46.02 vs. 5 ± 7.07, P = 0.02; 175.78 ± 114.12 vs. 62.5 ± 17.68, P = 0.001; 258.78 ± 203.7 vs. 125 ± 35.36, P = 0.02, respectively; **Table 3**). Patients with *Escherichia*-positive CRC consumed significantly higher amounts of processed and total meat than patients with *Escherichia*-negative CRC (29.5 ± 45.93 vs. 0 ± 0, P *=* 1.228e - 5; 260.5 ± 202.28 vs. 65 ± 49.49, P = 0.04, respectively; **Table 3**). Furthermore, the body mass indices (BMIs) of patients with *Streptococcus*-positive and *Streptococcus*-negative CRC were significantly different (27.4 ± 4.74 vs. 25.52 ± 3.16, respectively; P = 0.04; **Table 3**). Regarding the clinical characteristics with binomial values, *Streptococcus* was marginally associated with sex (P = 0.05; **Table 4**), whereas *Fusobacterium* was associated with tissue infiltration, although the difference in CTs was marginally significant (P = 0.11; **Supplementary Table S2**).

**Table 3:** Variations in epidemiological characteristics with continuous values based on the association of CRC with the four Ct values.

Bacteria	Relative abundance ≥1%	Age	BMI	Tumor size	CEA	Processed meat consumption, g/day	Red meat consumption, g/day	Total meat consumption, g/day
*Fusobacterium nucleatum*	Positive	61.36 ± 10.81	27.06 ± 4.60	4.75 ± 2.33	87.29 ± 371.32	31.64 ± 47.76	167.8 ± 109.68	252.74 ± 208.92
	Negative	64.81 ± 14.39	25.67 ± 2.78	3.27 ± 1.92	15.69 ± 25.48	8.75 ± 18.08	195.0 ± 141.15	269.5 ± 161.63
	P-value	0.46	0.18	0.04^*^	0.33	0.02^*^	0.59	0.78
*Bacteroides fragilis*	Positive	61.71 ± 11.47	26.97 ± 4.46	4.66 ± 2.28	NA	29.32 ± 46.02	175.78± 114.12	258.78 ± 203.73
	Negative	64.66 ± 5.77	24.31 ± 2.06	1.00 ± 0	NA	5.00 ± 7.07	62.50 ± 17.68	125.00 ± 35.36
	P-value	0.48	0.14	2.2e-16^*^	NA	0.02^*^	0.001^*^	0.02^*^
*Streptococcus gallolyticus*	Positive	61.60 ± 11.52	27.40 ± 4.74	4.55 ± 2.55	100.72 ± 402.03	25.44 ± 43.06	173.97 ± 118.37	243.30 ± 213.22
	Negative	62.39 ± 10.95	25.52 ± 3.16	4.60 ± 1.70	13.81 ± 22.66	34.25 ± 50.29	168.00 ± 107.96	281.81 ± 175.87
	P-value	0.77	0.04^*^	0.92	0.31	0.51	0.85	0.43
*Escherichia coli*	Positive	62.0 ± 11.37	26.89 ± 4.46	4.59 ± 2.35	NA	29.5 ± 45.93	176.05 ± 113.55	260.5 ± 202.28
	Negative	54.5 ± 4.95	26.24 ± 3.04	3.75 ± 0.35	NA	0.0 ± 0	55.00 ± 63.64	65.0 ± 49.49
	P-value	0.25	0.81	0.08	NA	1.228e-05^*^	0.19	0.04^*^

^*^Statistically significant at P < 0.05.

**Table 4:** Variations in epidemiological characteristics with binary values based on the association of CRC with the four Ct values.

Bacteria	Relative abundance ≥1%	Sex		Diabetes		Smoking		Alcohol		Hypertension		Tumor location		MSI		Nationality		Degree of different	
		M	F	Yes	No	Yes	No	Yes	No	Yes	No	Pro	Dis	Low	High	Asians	Europeans	GII	GIII
*Fusobacterium nucleatum*	Positive	35	37	12	60	14	58	2	69	33	39	23	49	44	6	47	24	27	45
	Negative	4	7	3	8	1	10	0	11	6	5	2	9	8	0	10	1	5	6
	P-value	0.53		0.41		0.68		1		0.75		0.49		0.58		0.159		0.74	
*Bacteroides fragilis*	Positive	38	42	15	65	15	65	2	77	33	37	25	55	51	6	55	24	30	50
	Negative	2	1	0	3	0	3	0	3	1	2	0	3	1	0	2	1	2	1
	P-value	0.61		1		1		1		1		0.55		1		1		0.55	
*Streptococcus gallolyticus*	Positive	24	36	13	47	12	48	1	58	30	30	18	42	35	5	40	19	24	36
	Negative	15	8	2	21	3	20	1	22	9	14	7	16	17	1	17	6	8	15
	P-value	0.05^*^		0.22		0.54		0.48		0.46		1		0.65		0.79		0.80	
*Escherichia coli*	Positive	39	42	15	66	15	66	2	78	39	42	25	56	50	6	56	24	32	49
	Negative	0	2	0	2	0	2	0	2	0	2	0	2	2	0	1	1	0	2
	P-value	0.49		1		1		1		0.49		1		1		0.519		0.52	

^*^Statistically significant at P < 0.05.

**Figure 4:**
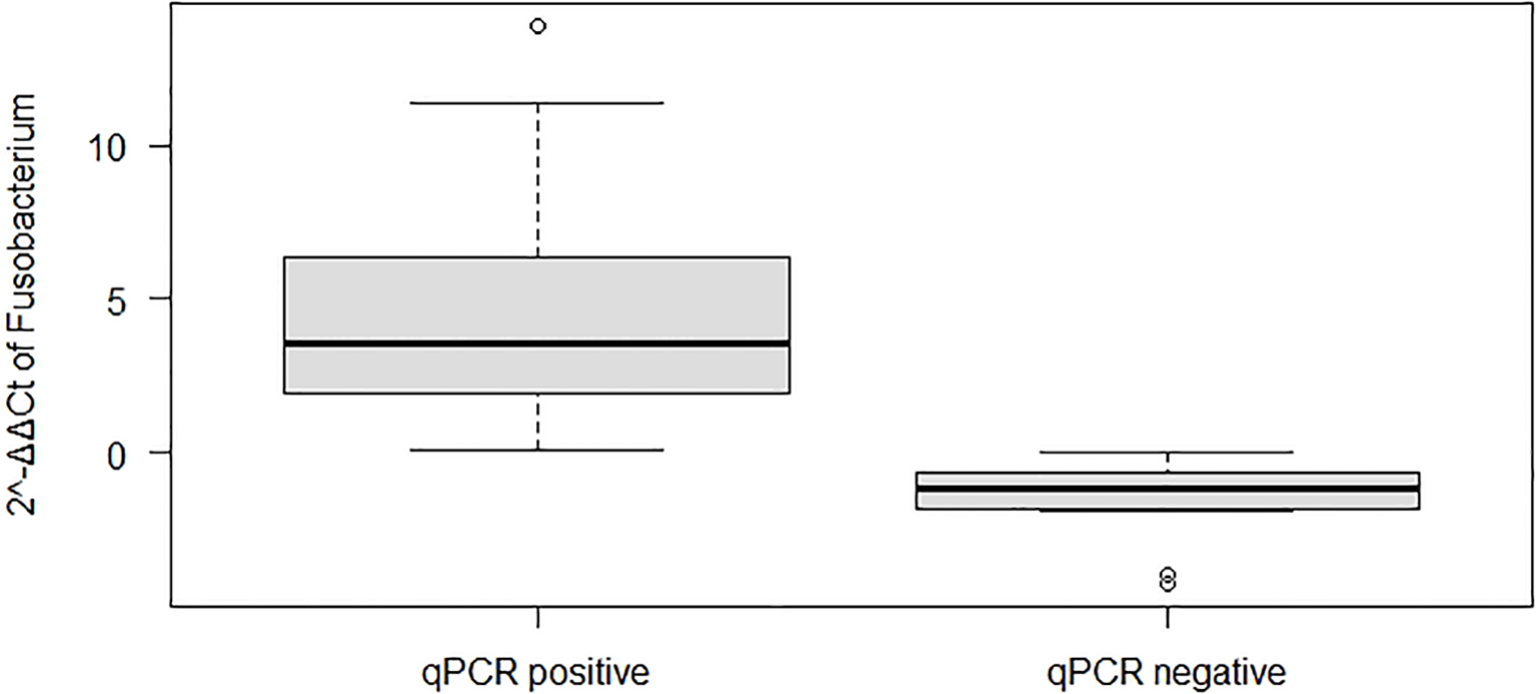
Boxplots showing the abundance of *Fusobacterium nucleatum* based on quantitative real-time polymerase chain reaction (qPCR) analysis and its positivity status.

The tumor sizes were significantly larger in patients with *F. nucleatum*-positive CRC than those in patients with *F. nucleatum*-negative CRC (P = 0.04; **Supplementary Figure S1A**). With respect to tumor location, the *F. nucleatum* Ct values (fold change) were significantly higher in the descending colon (P < 0.03; **Supplementary Figure S1B**) than those in other parts of the colon. The tumor stage was not significantly correlated with the presence of specific bacteria. Regarding CTs, comparison of *F. nucleatum* fold-change values (Ct values) revealed that qPCR-positive cases had higher fold-change abundance values than those of qPCR-negative cases, and these values were significantly differ (P < 0.05; **Figure 4**).

## 4 Discussion

Till date, the role of *F*. *nucleatum* in the development of CRC remains unclear. Determining the etiology of CRC can lead to the development of preventive and therapeutic strategies. In this study, we investigated the association between CRC, specific gut bacteria, clinical characteristics, and diet to determine the role of specific microbes in CRC development and the relationship between microbiota and red/processed meat in CRC. We determined the abundance of CRC-associated bacteria using qPCR and the statistical correlations between the clinical characteristics and outcomes of colon infections caused by the most common species that infect the colon. One of the important findings of our study is that among the infections caused by the four species analyzed, *F*. *nucleatum* infection is a serious and common infection. *F. nucleatum* has drawn attention for its possible link to CRC; however, infections caused by this species have been extensively studied and documented in case reports and large series of reports (20).

The gut bacteria play a significant role, and dysbiosis can lead to colonic carcinogenesis via chronic inflammatory mechanisms (21). Microbial dysbiosis can alter the host gene expression and inflammatory responses, creating a microenvironment that promotes cancer development. Several studies have shown a significant increase in the numbers of *F. nucleatum*, *S. gallolyticus*, *E. coli*, and *B. fragilis* in patients with adenomas or adenocarcinomas compared with those in healthy individuals (22).

Enterotoxigenic *B. fragilis* contributes considerably to the development and progression of CRC via its toxin-mediated effects on colonic cells and the immune system (23). Further, our study reported a 48.2% carriage rate of *B. fragilis* in patients with CRC using CT samples and a rate of 37.4% using ATs; and a 30.1% colonization rate was observed in normal tissue samples from patients with CRC, with a marginally significant difference between tissue types (P = 0.06; **Table 1**). However, we observed a marginally significant difference in the prevalence of *B. fragilis* between the CT and NT samples (P = 0.06; **Figure 3**), with the prevalence in CT (48.2%) being significantly lower than that reported in a previous study (86%) (24). A recent qPCR study revealed that only 6.1% of CRC cases tested positive for ETBF. Additionally, *B. fragilis* has been associated with good outcomes in patients with stage II and III CRC after curative resection (25). Another study found an association between fecal ETBF and CRC, with *B. fragilis* present in 58.3% of CRC cases compared to 26.6% occurrence of *B. fragilis* in controls (P < 0.05). Furthermore, the presence of *B. fragilis* in patients with stage III CRC was significantly higher than that in patients with stage I and II CRC (P < 0.05) (23). Therefore, further studies are required to determine the prevalence and distribution of ETBF.

Certain strains of intestinal *E. coli* can potentially affect the onset and progression of CRC by utilizing virulence factors and inflammatory pathways. Mucosa-associated *E. coli* strains are found more frequently in CRC biopsies than in healthy individuals (26). The *uidA* gene, which encodes beta-glucuronidase in *E. coli*, was used to determine total *E. coli* DNA concentrations (27). The presence of *clbB* gene a part of the *pks* island of *E. coli*, in patients with CRC might indicate an association between *E. coli* and CRC. Recent studies show that some *E. coli* strains possessing a gene cluster named the *pks* island might have a causative role in the development of human colorectal cancer (CRC). However, the results from the Japanese population showed that the prevalence of *pks*-positive *E. coli* was not significantly higher in CRC patients compared to controls (27). Deletion of the polyketide synthase (pks) genotoxic island from *E. coli* NC101 decreased tumor multiplicity and invasion in AOM/Il10−/− mice, without altering intestinal inflammation (28). Mucosa-associated pks+ *E. coli* was found in a significantly high percentage of IBD and CRC patients. This suggests that colitis can promote tumorigenesis in mice, by altering microbial composition and inducing the expansion of microorganisms with genotoxic capabilities (28). In our study, the prevalence of total *E. coli* in patients was significantly different between the CT and NT groups (P = 0.01; **Figure 3**). The frequency of *E. coli* among the tissue types was also significantly different (P = 0.04; **Table 1**). Our results indicate a potential relationship between total *E. coli* and CRC.

The prevalence of *S. gallolyticus* was investigated using qPCR with primers specific for superoxide dismutase (*sodA*) (29). Patients demonstrate higher levels of this bacterium than observed in healthy individuals (30). Colonic cells incubated with *S. gallolyticus* showed elevated levels of β-catenin, c-MYC, and proliferating cell nuclear antigen (PCNA), which are transcription factors linked to cancer development. Additionally, in mice, administration of *S. gallolyticus* results in a greater number of tumors, increased tumor burden, higher dysplasia grade, and enhanced cell proliferation and β-catenin staining in colonic crypts compared to that in mice treated with control bacteria (30). However, CRC-specific conditions such as elevated bile acid concentrations may also encourage *S. gallolyticus* colonization and perpetuate high levels of this bacterium in the gut. In our study, *S. gallolyticus* was not commonly found in patients with cancer (**Table 1**); however, *S. gallolyticus* was associated with BMI (**Table 3**).

Recently, *F. nucleatum* has gained attention as a potential cause of CRC (1, 14, 16). Although the role of *F. nucleatum* in CRC pathogenesis remains incompletely understood, four mechanisms have been proposed to explain its involvement. 1) Promotion of cell proliferation via WNT signaling through the interaction between *FadA* (adhesin A) and E-cadherin; *F. nucleatum* expresses proteins like *FadA* and *Fap2*, which allow it to adhere to host epithelial cells (31). *FadA* binds to E-cadherin on host cells, triggering β-catenin signaling pathways (32). Activation of the β-catenin signaling pathway through *FadA* binding leads to increased expression of oncogenes and enhanced WNT signaling, which promotes uncontrolled cell division and tumor growth (33). 2) Evasion of antitumor immune responses through the interaction of galactose-inhibitable autotransporter adhesion (Fap2) with T cell immunoreceptors containing immunoglobulin (Ig) and immunoreceptor tyrosine-based inhibitory motif domains (TIGIT); *F. nucleatum* uses the Fap2 protein to bind to TIGIT, an inhibitory receptor on T cells and natural killer cells (34). This interaction reduces immune surveillance, allowing cancer cells to evade detection. TIGIT has been linked to the exhaustion of natural killer cells and T cells in CRC. 3) Binding to tumors and increase in colonization through Fap2 and galactose-N-acetylgalactosamine (Gal-GalNAc) interactions; Fap2 interacts with Gal-GalNAc sugars on cancer cells, facilitating bacterial adhesion specifically in CRC tissues (35). After adhesion, *F. nucleatum* can penetrate epithelial cells, disrupting their integrity and contributing to the development of chronic infection. 4) Contribution to chemoresistance via lipopolysaccharide and toll-like receptor mechanisms (17, 18, 20). *F. nucleatum* induces chronic inflammation, which is a well-known driver of cancer. Lipopolysaccharide, a component of the bacterial cell wall, binds to TLR4 (Toll-like receptor 4) on immune and epithelial cells, triggering NF-κB signaling, leading to the production of pro-inflammatory cytokines such as IL-6, IL-1β, TNF-α, and IL-17 (36). These cytokines create a pro-inflammatory tumor microenvironment, promoting cancer cell proliferation, angiogenesis, and resistance to apoptosis (37).

*Fusobacterium* species are obligate anaerobes that pose challenges for isolation using culture methods. In this study, the prevalence of *F. nucleatum* in the CTs of patients analyzed using qPCR (43.37%) was significantly higher than that obtained using anaerobic cultures (9.6%; data not shown). Hence, non-culture-dependent detection techniques such as qPCR analysis could be crucial for screening *Fusobacterium* species or investigating its epidemiology in a population during CRC progression (38). The presence of *F. nucleatum* was significantly correlated with the location of CRC (**Table 2**) (39, 40). These findings suggest that *F. nucleatum* plays a role in the early stages of CRC development. One review suggested an association between *F. nucleatum* and carcinomas at various stages of CRC progression. Analysis of *F. nucleatum* abundance by tissue type indicated a higher prevalence of bacteria in CTs than that in ATs and NTs (**Figure 2B**). This observation aligns with those of previous studies indicating that elevated *Fusobacterium* colonization levels are associated with CRC (14). Certain microorganisms, such as *F. nucleatum* and *E. coli*, are prevalent in the colonic mucosa and have the potential to accelerate cancer progression and malignancy.

Conversely, diets high in red and processed meat have been associated with CRC development (41). However, the intricate metabolic and inflammatory mechanisms underlying the association between diet and cancer remain unclear. The primary carcinogenic factors associated with the consumption of red and processed meat include heme compounds, heterocyclic amines, nitrosamines, and undigested proteins (42). In addition to the direct carcinogenic effects, these molecules can alter gut microbiota, thereby influencing gene expression and disrupting colorectal epithelial cell homeostasis, which may promote the development of CRC (43).

This study had a few limitations. First, we did not incorporate innovative concepts of molecular science into the study design. Second, we did not include the gene clbB of *E. coli* in our study, which has a strong association with CRC. Longitudinal studies are needed to establish the association of pks-positive *E. coli* infection with colorectal cancer in our population. Overall, our results captured the primary characteristics of the Kazakhstani population. However, further large-scale studies are required to validate these findings.

In conclusion, our findings indicate that *F. nucleatum* is prevalent in CRC tissues and is present in different tissue types in both early and late stages of CRC. Moreover, we found a positive association between *F. nucleatum* abundance, tumor size, tumor location, and processed meat consumption in patients with CRC. The findings presented here emphasize the role of *F. nucleatum* in the tumorigenesis and progression of CRC. Further investigation is required to identify the genetic and phenotypic diversity of *F. nucleatum* colonizing tumors, which contribute to the initiation of CRC. No ethnic differences were observed with regard to this association. To the best of our knowledge, this is the first report on the association between *F. nucleatum* and CRC in Kazakhstani patients. Our findings suggest that *F. nucleatum* may be a potential marker for CRC diagnosis.

## Data Availability

The raw data supporting the conclusions of this article will be made available by the authors, without undue reservation.
