# Supplementary material for: Association of Fusobacterium nucleatum infection with colorectal cancer in Kazakhstani patients
Source: Front Oncol. 2024 Dec 12;14:1473575. doi: 10.3389/fonc.2024.1473575 (PMC11669545; doi:10.3389/fonc.2024.1473575)
Supplement: Supplementary file 1 [file DataSheet1.docx]

Supplementary Material

# Supplementary Data

Supplementary Material should be uploaded separately on submission. Please include any supplementary data, figures and/or tables.

# Supplementary Figures and Tables

Table 1. Clinical features in Kazakhstani patients with colorectal cancer

| **Clinical and molecular features** | **n (%)** |
| --- | --- |
|  |  |
| Total number of patients  Total number of biopsies  Gender  Male  Female  Age of onset  <65  ≥65  BMI  <25  ≥25  Tumor size (cm)  <5  ≥5  Unknown  Alcohol  Drinker  Nondrinker  Unknown  Smoking  Ever  Never  Diabetes  Yes  No  Hypertension  Yes  No  Tumor location  Distal colon  Proximal colon  Disease stage  I  II  III  IV  MSI status  MSS/MSI-low  MSI-high  Unknown | 83  249  39 (47.0)  44 (53.0)  45 (54.22)  38 (45.78)  27 (33.75)  56 (66.25)  48 (57.83)  32 (38.55)  3 (3.62)  2 (2.41)  80 (96.34)  1 (1.20)  15 (18.07)  68 (81.93)  15 (18.07)  68 (81.93)  39 (46.99)  44 (53.01)  58 (69.88)  25 (30.12)  11 (13.41)  34 (41.46)  36 (43.9)  1 (1.22)  52 (62.65)  6 (7.23)  25 (30.12) |

Table 2. Differences in epidemiological characteristics with binomial values based on the association of CRC with the four Ct values.

| **Genus** | **Relative abundance ≥1%** | **Tissue infiltration** | | **Lymph node metastasis** | | **Stage** | | | | | | |
| --- | --- | --- | --- | --- | --- | --- | --- | --- | --- | --- | --- | --- |
|  |  | **T1+T2** | **T3+T4** | **N0** | **N1+N2** | **I** | **II** | | **III** | | **IV** | |
| *Fusobacterium* | Positive | 11 | 60 | 38 | 34 | 8 | 29 | | 33 | | 1 | |
|  | Negative | 4 | 7 | 8 | 3 | 3 | 5 | | 3 | | 0 | |
|  | P-value | 0.11^*^ | | 0.331 | | 0.337 | | | | | | |
| *Bacteroides* | Positive | 14 | 66 | 44 | 36 | 11 | | 33 | | 35 | | 1 |
|  | Negative | 1 | 1 | 2 | 1 | 0 | | 1 | | 1 | | 0 |
|  | P-value | 0.334 | | 1 | | 1 | | | | | | |
| *Streptococcus* | Positive | 12 | 47 | 33 | 27 | 8 | | 24 | | 26 | | 1 |
|  | Negative | 3 | 20 | 13 | 10 | 3 | | 10 | | 10 | | 0 |
|  | P-value | 0.539 | | 1 | | 1 | | | | | | |
| *Escherichia* | Positive | 15 | 65 | 45 | 36 | 11 | | 33 | | 35 | | 1 |
|  | Negative | 0 | 2 | 1 | 1 | 0 | | 1 | | 1 | | 0 |
|  | P-value | 1 | | 1 | | 1 | | | | | | |

## Supplementary Figures

**
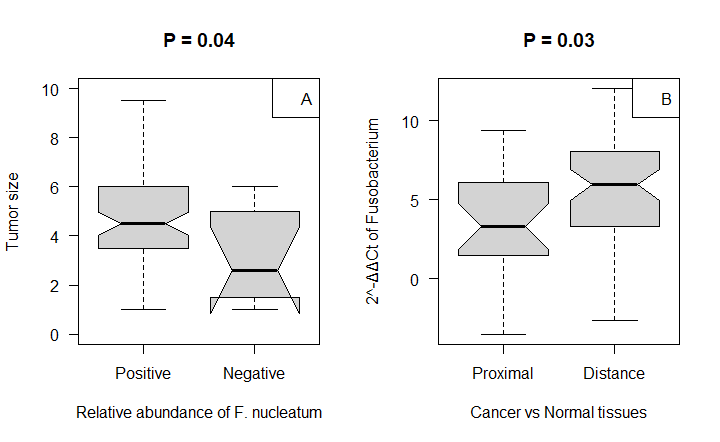
**

Figure 1. **(A)** Boxplots of the tumor size of patients between positive and negative statuses of the relative abundance of *F. nucleatum*. **(B)** Boxplots of the fold change abundance of *F. nucleatum* between tumor locations.
